# Supplementary material for: Are carbon emissions trading and green financial instruments synergistic? -Comprehensive quantitative research based on content analysis
Source: PLoS One. 2024 Mar 7;19(3):e0298601. doi: 10.1371/journal.pone.0298601 (PMC10919601; doi:10.1371/journal.pone.0298601)
Supplement: S1 Data — (ZIP) [file pone.0298601.s001.zip › Data/地级市Z_F工作报告.zip!/╔╜╢1⁄2/╡┬╓▌╩╨/└┤╫╘╣1⁄2╓┌║┼í╛┬φ┐╦╩2╛▌═°í┐_2015.docx]

各位代表：

现在，我代表市人民政府向大会报告工作，请予审议，并请市政协委员和其他列席会议的同志提出意见。

2014年主要工作回顾

过去一年，在中共德州市委的坚强领导下，在市人大、市政协及社会各界的监督支持下，我们紧紧依靠全市人民，坚持稳中求进、进中求好、好中求快主基调，稳增长、调结构、抓改革、惠民生，较好完成了市十七届人大四次会议确定的年度任务。

(一)经济规模质量实现同步提升。科学应对复杂形势，积极克服下行压力，巩固了“稳”的基础，保持了“进”的态势，实现了“好”的突破。主要经济指标平稳较快增长。实现地区生产总值2596.1亿元，增长10%，高于全省1.3个百分点；公共财政预算收入171.3亿元，增长14.2%，高于全省4个百分点；全社会固定资产投资1961.3亿元，增长16.3%，居全省第一；完成进出口总额35.1亿美元；城镇居民人均可支配收入27180元、农民人均纯收入12135元，分别增长9.5%和11.6%。经济结构更趋合理。三次产业比例调整为11.1∶51.6∶37.3，三产占比提高1.3个百分点；公共财政预算收入占GDP、税收占公共财政预算收入、主体四税占税收的比重，分别达到6.6%、81.3%和54.4%；县域经济“三年倍增”计划圆满完成，齐河县、德州经济技术开发区、德城区公共财政预算收入突破20亿元，齐河县跻身全国百强县序列。现代农业稳步发展。粮食生产实现“十二连增”，总产达173.7亿斤，连续三年荣获“全国粮食生产先进单位”称号；新增蔬菜面积18万亩，发展食用菌520万平方米，新建、提升标准化养殖场294家；新培育农民合作社1663家、家庭农场438家；综合机械化率达到91.2%，居全省首位；农村改革不断深化，85%的村完成土地承包经营权确权颁证；新增土地流转面积69.2万亩，被确定为国家级现代农业示范区。工业运行稳中向好。规模以上工业企业达3444家，居全省第4位，实现增加值1708.6亿元、利税958.1亿元，分别增长14%和10%；实施重点技改项目338个，完成投资336亿元，企业装备水平和发展动力明显提升。服务业全面提速。全市实现社会消费品零售总额1116.8亿元，增长12.8%；济铁物流园、银座财富中心、中关村产业园等一批高端项目加快建设，泉城欧乐堡梦幻世界、黄河故道温泉度假村、四女寺风景区、冀鲁边区革命纪念园等文化旅游景区开放运营，第三产业发展进入载体建设和业态创新的新阶段。

(二)十大产业集群培植取得重大进展。坚持一手抓存量优化、一手抓增量扩张，大力实施科技金融产业融合创新，积极推动要素驱动向创新驱动转变，努力打造德州产业升级版。产业招商成效明显。深入推进“一区一圈一带”战略，积极融入京津冀协同发展规划，先后举办了首都经济圈投资洽谈会、第四届太博会暨首都企业家德州论坛等系列招商推介活动，积极对接央企强企和全国工商联执委企业，全市新注册项目530个，新开工460个，新投产330个，亿元以上项目到位资金590亿元，张江高科示范园、中欧产业园等项目签约，威讯半导体、天津农垦示范园、祥辉铜业等重大招商项目建设进展迅速，安凯车桥、迈宝赫等重点财源项目竣工投产。106个市级重点项目完成投资472亿元，占年度计划的102%。企业培植实现突破。华鲁恒升、永锋钢铁、恒源石化、星光集团4家企业主营业务收入过100亿元，通裕重工、中大贝莱特、谷神生物、景津集团等15家企业过50亿元，55家科技型成长企业单体平均增长21.3%。民营经济加快发展，工商登记制度改革激发市场活力，实有市场主体总量达到23.9万户，同比增长17.1%。载体建设加快推进。47个工业产业基地主营业务收入占全市比重达到48.1%，生态科技城总体规划、基础设施建设基本完成，德州高新区晋升国家级通过科技部审核，7个科技创新创业园、5个高新技术产业园集聚引领作用更加明显，应用技术研究院完成规划设计。科技金融驱动力度加大。成功举办京津大院大所对接会等活动，签订分支机构建设协议138项；新增省级以上科技创新平台23个、高新技术企业31家，承担国家、省重大科技项目74项，实施科技成果转化项目286项，高新技术产业产值占比提高1.4个百分点；资本交易大会影响力不断扩大，引进和新设立金融机构57家，市内银行新增信贷投放243.6亿元，直接融资258亿元；设立5亿元的市级股权投资引导基金，政府扶持产业发展方式实现了重大转变；52家企业完成股改，6家企业在“新三板”挂牌；不良贷款率下降至2.55%，政府综合金融服务机构实现县域全覆盖。

(三)城乡建设迈出坚实步伐。在政策环境发生重大变化的情况下，积极创新工作思路，拓宽融资渠道，确保既定建设项目平稳有序推进。全市城镇化率达到49.5%，提高1.8个百分点，成功获批国家新型城镇化综合试点。中心城区承载能力提升。行政区划调整实现新突破，陵县撤县设立陵城区，主城区面积增加到1752平方公里，扩大3倍以上；全年实施6大类66个城建项目，完成投资159亿元；大外环、“两河四岸”滨河路全线贯通，新建改造道路23条，袁桥、项目东区南部和北部3个大社区竣工；华能德州电厂至高铁新区远距离供热管网和第四净水厂项目竣工运行，被列为全国首个城市地下管线综合管理试点城市。县城和重点镇建设加快。各县城共实施扩容提质建设项目308项，完成投资109亿元；15个省、市示范镇建成和在建项目174个，完成投资13.5亿元。生态文明乡村建设和“两区同建”稳步推进。新建续建新型农村社区216个、农村产业园区150个，建设垃圾转运站152座。基础设施建设有序实施。济乐高速通车运营，德大铁路、石济客运专线、德上高速建设进展顺利，新建改造农村公路1480公里；开工建设6座平原水库，完工4座，新增库容3236万立方米，全国小型农田水利重点县实现全覆盖。生态建设成效显著。三年大绿化任务全面完成，森林覆盖率达到35%，黄河故道森林公园和徒骇河、黄河水乡、九龙口3处湿地公园成功晋升国家级；深入开展环境污染治理，建成453个水污染防治工程，海河流域水污染防治专项考核荣获全国“双第一”；集中开展城市扬尘、汽车尾气、餐饮油烟等综合整治，完成18家重点企业限期治理，淘汰黄标车及老旧车29447辆、落后生产线25条、设备803台(套)，顺利完成节能减排年度任务。

(四)民生保障再上新水平。坚持问题导向，突出民生优先，保基本、兜底线、促均衡、办实事，一批民生问题得到有效解决。全年重点民生领域财政支出171亿元，占财政总支出的比重达到62%。年初确定的民生实事全部完成。全市开工建设中小学84所，新建、改造幼儿园289所，补充教师1503人，德州学院扩建工程进展顺利，华宇工学院升格为本科院校，职教公共实训中心和市人民医院新门诊楼扩建工程启动建设，第二批县级公立医院综合改革试点稳妥推进。新增城镇就业7万人，农村劳动力转移就业8.6万人；城镇居民医疗保险与新农合实现并轨，城乡居民大病保险全面实施，居民养老保险基础养老金标准提高到75元，企业退休人员月均增加养老金245元、实现“十连涨”，工伤、失业保险待遇明显增长；4处县级社会福利中心投入使用，新建城市社区日间照料中心38个，新增养老床位6324张。城市低保标准由每人每月300至320元提高到360至380元，农村低保标准由每人每年2000元提高到2400元，五保集中供养、分散供养标准分别由每人每年3500元、2400元提高到4000元、3000元；开工安居工程27834套，基本建成19200套。群众性文体活动丰富多彩，优化提升农村文化大院700家，建成村居文体小广场4038个，投资1100万元为926个乡村安装了健身设施；市县乡村四级文化场馆和设施实现免费开放，大剧院演出效果良好，文化精品创作和竞技体育比赛取得好成绩；文化遗产保护实现新突破，大运河成功申遗，德州扒鸡制作工艺和黑陶烧制技艺列入国家级非物质文化遗产名录，顺利通过国家二类城市语言文字评估。立体化社会治安防控体系不断健全，科技强警成效凸显，重大安保运行规范有序，反恐维稳、矛盾纠纷调处机制进一步完善，社会保持和谐稳定。食品药品监管有力，安全生产形势平稳，未发生较大及以上安全事故。双拥共建、国防动员、人民防空、民族宗教、老龄助残、妇女儿童等社会事业成效明显，防震减灾、应急管理、司法仲裁、外事侨务、对台事务、口岸打私、档案、史志、气象等工作取得新进展。

(五)政府运行更加规范高效。紧紧围绕为民务实清廉主题，扎实开展党的群众路线教育实践活动，有效整治解决了一批“四风”问题和群众反映强烈的热点问题，健全完善了优化营商环境、联系服务群众、改进文风会风等45项制度。主动接受人大、政协监督，充分吸纳运用人大代表、政协委员的意见建议和调研成果，将新型城镇化、旅游业发展、物业管理、学前教育等重要调研视察报告意见，及时转化为政府工作安排，办理人大代表建议和政协提案816件，办结率100%。建立政府法律顾问制度，聘任10名政府法律顾问。积极稳妥推进政府机构改革，食品药品、卫生计生、工商、质监管理体制改革基本完成。深化行政审批制度改革，积极推进电子政务和政府信息公开，创新“四证联办”工作机制，编制完成市级行政审批事项目录，取消、下放和转变管理方式164项，压减审批环节327个 金庸小说，成为全省行政审批事项最少、办理时限最短的市之一。市政务服务中心办理各类审批服务事项145万件，增长22%；公共资源交易成交额突破200亿元，增长30%。每季度集中研究“12345”市民热线反映的热点难点问题，全年受理有效诉求64.8万件，热线服务满意率98%，回访满意率88%，涉及部门职能交叉的17类民生难点问题得到妥善解决。强化政府投资项目资金评审和绩效管理，市级“三公”经费支出同比下降35.3%，公务接待下降47.2%。加强政府性债务管理，有效防范和化解了潜在财政风险。严格落实党风廉政建设责任制，深入实施廉政效能管理工程，强化经济责任审计，加大案件查处力度，政府廉政建设取得新成效。

各位代表，一年来的发展实践告诉我们：推进科学跨越发展，必须准确把握形势，构筑战略优势。注重战略谋划，积极对上争取列入京津冀协同发展规划和国家新型城镇化、现代农业示范区、农村土地管理制度改革、土地增减挂钩试点等一批事关德州长远发展的全局性事项，拓宽了大开放、大合作、大发展的空间。推进科学跨越发展，必须加快转调创步伐，实施创新驱动。找准做大德州实体经济的新路径，深入研究实施建基地、搭平台、引人才、设基金等新举措，打通科技金融产业融合发展的新通道。推进科学跨越发展，必须坚持问题导向，持续改善民生。着力解决总量不足、布局不均两大矛盾，兼顾需要与可能，注重均衡，分步实施，多元投入，增加供给，兜住底线，真正提升群众幸福感、满意度。推进科学跨越发展，必须大力简政放权，优化营商环境。突出权力清单式管理，突出讲规矩重程序，突出提效率转作风，激发全社会创新创业活力。

各位代表，德州今天良好的发展局面，是上级党委、政府和市委正确领导的结果，是市人大、市政协和社会各界大力支持的结果，是全市人民团结奋斗、拼搏实干的结果。在此，我代表市政府，向全市人民，向各位人大代表、政协委员、离退休老同志，向各民主党派、工商联、人民团体和各界人士，向驻德部队官兵，向中央、省驻德单位，向关心支持德州发展的海内外朋友，致以崇高的敬意并表示衷心的感谢!2015年总体安排和重点工作

今年是全面完成“十二五”规划的收官之年，是全面深化改革的关键之年，是全面推进依法治市的开局之年，也将迎来有纪念意义的撤地设市20周年。德州的发展，既面临严峻挑战，更面临难得机遇，必须以战略的眼光洞察形势，以创新的思维谋划工作，以务实的举措推进落实。

新形势新挑战，需要我们认清差距、加压奋进。当前，我市实体经济发展不足、产业层次较低、部分企业经营困难的阶段性矛盾仍然突出；城乡发展不均衡，城镇功能不健全，承载力、辐射力不强；教育、医疗等公共服务资源与群众需求还有差距；政府服务效能、工作执行力、担当意识更需大幅提升。我们要强化底线思维，变压力为动力，化挑战为机遇，在战胜困难中取得更大突破。

新常态新作为，需要我们高点定位、转型发展。经济发展进入新常态，正在向形态更高级、分工更复杂、结构更合理的阶段演化。中央确定了“稳中求进”的总基调，更加注重质量效益、结构调整、改革创新、风险防控、民生保障。我市处在新型工业化、城镇化加速推进的关键时期，面临着宏观政策趋紧、资源环境制约的双重难题，做大经济总量、加快转型发展的双重考验。我们既要积极适应新常态，又要立足德州实际，努力做到调整不减速、量增质更优，促进经济持续平稳健康发展。

新时期新优势，需要我们抢抓机遇、乘势而上。京津冀协同发展规划初步定位我市为“四基地一功能区”，带来新的政策红利。43项国家和省级试点赋予我们先行先试的巨大优势。同时，近年来大开放、大建设积蓄的势能加速释放，科技金融产业融合创新催生新的增长点，陵县撤县设区拓展了城市空间，一批重大项目提升了发展后劲，经济运行在较快增长区间的基础依然牢固，完全有条件、有能力把良好势头保持下去。

根据上级要求和市委决策部署，综合分析各方面因素，今年政府工作的指导思想是：全面贯彻落实党的十八大和十八届三中、四中全会精神，以习近平总书记系列重要讲话特别是对山东工作的重要批示精神为指导，继续坚持稳中求进、进中求好、好中求快主基调，主动适应经济发展新常态，突出扩量提质增效并举，着力转变工作指导方式，坚持改革攻坚、创新驱动、区域合作、依法行政，加快推进产业集群化、新型城镇化、农业现代化、民生均等化，努力完成“十二五”规划目标，在科学跨越发展、建设幸福德州进程中干在实处、走在前列。

全市经济社会发展的主要预期目标是：地区生产总值增长9%，公共财政预算收入增长11%，固定资产投资增长16%，社会消费品零售总额增长12%，外贸进出口增长5%，实际利用外资平稳增长，城镇居民人均可支配收入和农民人均纯收入分别增长10%、11%，城镇化率达到51%，城镇登记失业率控制在3.5%以内，居民消费价格涨幅控制在3%左右，人口自然增长率控制在10.4‰以内，节能减排指标确保完成省下达任务。

实现今年总目标，重点抓好以下六方面工作：

(一)突出科技金融产业融合创新，加快提升产业实力。围绕打造京津冀产业转移承接基地和科技创新成果转化基地，集聚要素资源，突破关键领域，培植壮大十大产业集群，把德州发展根基建立在坚实的实体经济之上。

抓技改、促升级。以技术改造为载体，推动企业加快向产业链、价值链高端延伸，实施重点技改项目350个，总投资410亿元。深入调查研究，掌握技改需求，解决好“谁需要技改”的问题。明确主攻方向，以新技术、新工艺、新装备、新产品推广应用为重点，解决好“围绕什么技改”的问题。加强资源配置，针对企业技改涉及的资金、技术、人才等支撑条件，搞好对接谋划，实现技改与市场形势、现实条件的有机结合，解决好“靠什么技改”的问题。正确处理政府与市场关系，引导60家骨干企业和55家科技型成长企业站在市场前沿，细化项目评估论证，整合各类资金，用好扶持政策，解决好“怎么推动技改”的问题。

抓梯队、壮群体。实施好“四个一批”工程，加快形成企业梯次发展格局。培育一批大企业集团，通过拉长产业链条、并购重组、强强联合等方式，推动重点企业倍增式膨胀。全力抓好华鲁恒升、实华化工等投资过百亿的重大项目，力争用2—3年时间，培植出3—5家销售收入过200亿的大企业集团。壮大一批行业龙头企业，开展对标提升行动，找准发展定位和赶超目标，谋划支撑项目和要素保障，加快培植景津、金能、索通、谷神、玲珑、保龄宝、金麒麟等企业，增强行业话语权。做强一批高精尖优势企业，围绕新材料、新能源、电子信息、交通装备、民用航空、医药医疗等重点领域，推进威讯半导体、德法尔电子、信立泰药业等高新技术企业加速扩张，尽快把技术优势变为规模效益优势。提升一批中小微企业，认真落实促进民营经济发展的政策措施，积极培育市场主体，开展中小微企业“政保贷”，鼓励大众创业、万众创新，实施回乡创业工程，推动个体工商户向小微企业发展，小微企业向规模企业提升。

抓招商、扩增量。实施“招商引资转型突破年”活动，适应国家调整招商优惠政策的新形势，运用市场机制，创新合作形式和支持途径，分产业、分领域制定招商计划，以龙头企业、重大项目为核心，加快引进缺失链条、补强薄弱链条、提升关键链条，力争全年亿元以上项目到位资金达600亿元。加强与央企、强企、军企、外企对接，充分借助中国企业联合会等优势资源，精心组织招商推介活动，建立重点客商清单制度，引进一批战略支撑力强的大项目。突出交通、资源、生态三大优势，拓展区域合作领域，深入推进“一区一圈一带”战略，深度融入京津冀协同发展区，积极落实与北京、上海、天津、台湾共建飞地园区的协议。完善招商项目跟踪对接、审批服务、落地建设等机制，加快芭蕾雨奥特莱斯、广药大健康基地等项目开工建设。持续抓好市级重大项目，完成投资400亿元。

抓载体、促集聚。把县域经济作为主要板块，启动实施新一轮县域经济提升计划，力争通过3—5年的努力，各县(市、区)公共财政预算收入在2011年基础上实现第二个翻番。搞好“开发区改革创新二次创业年”活动，推动投融资体制、管理模式和政策环境创新，主要解决产业总量不够、密度强度不够、集中度关联度不够、配套支撑不够等问题，争取德州高新区、齐河经济开发区晋升国家级；创新园区开发模式，支持发展飞地经济，引导企业、项目向园区集中布局。按照特色鲜明、错位发展的要求，加快47个工业基地建设，以龙头企业为依托，以产业配套合作为基础，增强项目选择与园区定位契合度，打造功能现代化、产业高端化的工业聚集区。充分发挥海关口岸、检验检疫等部门职能作用，提升青岛保税港区(德州)功能园区载体优势，积极申建德州综合保税区，推动外经贸转型升级。大力实施企业“走出去”战略，充分利用国际国内两个市场、两种资源，在境外投资创业、建设生产基地、合作开发等方面实现新的突破。

抓业态、强功能。在抓好传统业态升级的同时，大力拓展新经济业态，培育新商业模式，提升拉动实体经济的能力。发挥新能源产业基础优势，加速提升档次、塑造品牌、扩张规模，尽快形成具有区域竞争力和影响力的产业集群 侯卫东官场笔记。抓住加快发展体育产业上升为国家战略的机遇，以宁津、乐陵、庆云为核心板块，壮大体育产业，促进体育消费。鼓励发展楼宇经济，集聚金融、保险等新兴服务业。围绕建设区域性商贸中心，支持大型商贸企业做大做强，培育大型物流企业，增强城市商业服务功能，打造全国性流通节点城市。大力发展网络交易市场和电子商务，实施“六进”工程，落实“十百千万”计划，引进知名龙头企业，发展淘宝特色产业村，使网络经济成为新的增长点。

抓创新、增后劲。把创新驱动作为经济内生增长的新引擎，努力创建全国第二批促进科技和金融结合试点城市、省科技金融产业融合创新综合试验区。完成市应用技术研究院一期主体建设，建立科技金融产业融合创新服务中心，落实京津大院大所对接成果，组织好京津、长三角大院大所科技成果对接会、山东省军民科技融合项目对接会、院士专家德州行等招研引学活动，抓好科技创新创业园、高新技术产业园和企业工程(技术)中心、院士工作站、博士后科研工作站、重点实验室建设，力争高新技术产业产值占比达到30%以上。运作好市级股权投资引导基金，设立齐鲁创新、新兴产业发展担保、科技成果转化等专项扶持基金，设立科技支行，建设股权交易市场，增强资本交易大会融资功能，完成股改企业100家，新增上市挂牌企业10家，力争全年市内银行新增信贷投放260亿元，直接融资300亿元。深入实施现代产业人才支撑计划和“金蓝领”培训工程，加大高端人才培养引进力度，建设人才公寓，加强知识产权保护，建设100万平方米科技企业孵化器，引进培育100个研发创新团队，形成创新人才集聚高地。

(二)突出城乡统筹，加快推进新型城镇化。以四级城镇体系建设为抓手，积极构建城乡协调、分工合理、功能互补的城镇格局。

以行政区划调整促进中心城区融合发展。围绕区域性中心城市建设，加快修编城市总体规划，合理布局老城、新区、生态科技城和陵城区，推动一体化、差异化发展。编制完成中心城区重点区域设计方案，完善控制性详细规划和专项规划，科学规划城市“三区四线”。加快疏解老城功能，增强新区吸纳承载能力，搞好城市设计研究和品牌形象策划，精心打造特色街区、重点路段、关键部位。加快生态科技城建设，坚持产城融合，规划建设杨庄河科技研发带，完善教育、医疗、商贸、旅游等服务设施，提升聚产业、聚人气的能力。加快陵城区融入主城区步伐，推动产业发展高端化、城市建设特色化、民生事业同城化，打造功能完善、配套齐全、生态宜居的增长极。实施城市组团发展战略，加快推进武城、平原、宁津与中心城区融合发展，促进基础设施、产业布局、资源要素、公共服务互补对接。

以公共资源优化配置提升城乡人居环境。坚持以人为核心的新型城镇化，持续性、长效性、均衡性地配置公共资源。中心城区今年计划投资153.2亿元，推进6大类、83项城建工程。加快德州外滩、“两河四岸”绿化等景区生态建设工程，打造城市景观核心区和休闲观光带；推进老工业区、老商贸区、棚户区和城中村搬迁改造，抓好大社区、保障性住房建设，有序开展“三无小区”改造和幸福社区创建，扎实做好便民市场建设、公厕改造、环卫设施完善等工作，确保顺利通过国家卫生城市复审；实施广川大道北延、减河堤顶路南延等城市道路新建、改造工程，打开断头路，疏通拥堵点；开展地下管线综合管理试点，合理开发利用地下空间。健全城市管理体制，强化精细化管理，加强规划管控，实施违章建筑大清理行动。加大城乡结合部、城市出入口等重点部位环境综合整治力度，塑造整洁靓丽的城市形象。有序推动县城扩容提质，增强服务功能，提升带动能力。加快国家、省、市示范镇建设，搞好功能定位，完善配套设施，努力建成一批文化旅游、商贸物流、资源加工、交通枢纽等特色名镇。坚持“两区同建”和文明乡村建设相结合，根据基础条件、群众意愿和发展需要，提升产业园区就业富民能力和居住社区配套功能，新建农村社区30个、续建150个，新建扩建农村产业园区100个，让农民群众增收有保障、生活有改善。

以基础设施建设带动城乡一体发展。发挥基础设施对城镇化的拉动作用，突出环境整治和城乡改貌，提升城镇化质量。推动城乡交通一体化，重点实施G308、G309、S246、S247、S249、S324道路改造，新建、改造农村公路900公里，抓好石济客运专线及高铁东站房、济齐黄河大桥建设，争取德上高速公路、德大铁路通车运营，超前谋划通用航空发展，规划实施中心城区快速公交通道，提升公交现代化水平，加快构建贯穿城乡的综合交通网络。推动供水、供热、供气等基础设施建设，重点抓好供水一体化，让城乡居民喝上同源、同质、同网的放心水，创建全国农村饮水安全和水利建管改革创新示范市。推动城乡环卫一体化，实行垃圾户集、村收、镇运、县处理。推动公共服务一体化，促进文化教育、医疗卫生、体育健身、就业创业、社会保障等服务功能均衡布局，缩小城乡差距。推动户口登记管理一体化，深化户籍制度改革，有序实现常住人口市民化。

(三)突出增效富民，大力推进农业现代化。以国家级新型城镇化和现代农业示范区“双试点”为契机，以建设京津优质农产品供应基地和高素质劳动力培训输送基地为依托，深化农村各项改革，转变农业发展方式，加快构建现代农业产业体系，走产品安全、产出高效、资源节约、环境友好的现代农业发展道路。

积极培育新型经营主体，促进农业经营规模化。基本完成农村土地承包经营权确权登记颁证工作，做大做强专业大户、家庭农场、农民合作社、农村专业技术协会等新型农业经营主体，新发展农民合作社500家、家庭农场150家；深化供销社改革，新增农村现代流通服务网点1000家。强化农业科技支撑，积极推广先进技术和绿色增产模式，构建育繁推一体化的现代种业体系。大力发展社会化服务，重点支持发展病虫害统防统治、农资供应、农产品储运保鲜、气象防灾减灾等专业化服务。加强农田基础设施建设，新增“旱能浇、涝能排”高标准农田113万亩。大面积推广深耕深松、配方施肥、减量投放等农地改良措施，提高农田质量。积极扶持发展农机合作社，耕种收综合机械化水平达到92%以上。

加快内部结构调整，促进农业生产集约化。在继续抓好高产创建、稳定粮食面积基础上，加快由“生产导向”向“消费导向”转变，大力发展优质特色精品农业。积极培育优质蔬菜、林果苗木、良种繁育、水产养殖、畜产品等高效特色农业，新增设施蔬菜15万亩、花卉苗木3万亩，发展特色渔业基地19万亩，新建、提升标准化规模养殖场200处以上，创建国家畜牧产业化循环发展示范区。抓好出口农产品质量安全示范市和“粮安工程”建设，推进农业标准化生产和质量监管，确保农产品抽检合格率稳定在96%以上。实施农产品品牌培育工程，力争“三品一标”产品产地认定面积占比达到60%以上。

不断拓宽增收渠道，促进农民收入多元化。大力推动农业产业化，促进产加销、农工贸一体化融合发展，新增农业龙头企业30家，扩大就近就业规模，增加农民工资性收入比重。实施新型职业农民培育工程、新型农民科技和创业培训工程，全年培训农民20万人次，农村劳动力转移就业8.5万人，提高农民农业收入和非农收入水平。开展农村土地管理制度改革试点，建立农村产权交易市场，努力增加农民财产性收益。认真落实各项支农惠农政策，盘活农村集体产权资源，做大做强集体经济，提高农民分配性收益。(四)突出宜居宜业，加快生态环境建设。坚持减量化排放、循环化生产、资源化利用，深入实施碧水、蓝天、绿地、宁静、整洁五大工程，发展低碳产业，建设生态城市，促进绿色消费，打造京津南部生态功能区。

抓好城乡生态绿化。以建设国家森林城市为目标，增加城市绿地和城乡林地面积，高标准实施鲁冀边界防护林带建设，对铁路、高速公路、干线道路、河流水渠沿线进行补植修复，全年新植树木1500万株，造林20万亩。巩固国家园林城市创建成果，完善城市绿地系统，实施街头绿地、公园绿地、道路景观、环城绿带等改造提升工程，完成绿化110万平方米。

大力实施蓝天行动计划。大气污染防治是重大民生问题，必须治标治本多管齐下。以控煤、控尘、控烟气为重点，深化重点行业大气污染监督管理，加强重点点源达标治理，完成14家燃煤电厂脱硝设施升级改造。完善环境空气质量生态补偿制度。淘汰黄标车及老旧车1.8万辆，加强工业废气、城市扬尘、油气回收治理，力争空气质量同比改善5%。

强化水污染防治。完善“治、用、保”治污体系，健全人工湿地、污泥处置、污水处理等治污工程运行管理机制，推进城镇污水集中处理设施建设，确保58个重点乡镇、社区污水处理设施正常运行。开展“环保五型企业”创建活动，巩固主要河流治污成果，着力抓好支流水质改善，实现主要河流断面水质稳定达标。加强饮用水源地保护，保障全市人民饮水安全。

加强资源节约利用。积极发展节能产业，严控主要污染物排放，严把环境准入条件，严禁新上高污染、高耗能项目，加快淘汰落后产能。抓好重点领域节能，推进新能源示范城市建设，规范太阳能和地源热泵应用管理。鼓励节能减排技术开发与推广应用，大力实施一批循环经济、清洁生产和节能降耗示范工程。支持平原县创建全国循环经济示范县。开展土地节约集约利用试点工作，完善市场配置机制，加强土地批后监管，加大闲置和低效利用土地清理整治力度，提高土地利用效率。

(五)突出民生优先，不断深化幸福德州建设。按照增加供给、守住底线、优化布局、科学管理、提高水平的要求，强化问题导向，持续加大投入，认真实施好年度民生实事，多做保本兜底的工作，多做雪中送炭的事情，在包容性发展中建设幸福德州。集中力量组织实施“六大民生工程”：

就业创业提升工程。就业是民生的首要问题。以解决结构性失业和高校毕业生就业为重点，认真落实创业带动就业扶持政策，整合各类创业培训资源，抓好创业孵化基地、创业园区和各级人力资源市场建设，全年组织开展就业创业培训6万人次，新增城镇就业6万人。

城乡教育提升工程。新建、改扩建中小学80所、学生食堂200处，改造薄弱学校100所，大力提升中小学校舍标准化水平。建设省级标准化幼儿园70所，新增幼儿学位1.2万个，逐步解决优质教育资源供给不足和布局不均问题。加快高级师范学校、德州二中规范化改造等项目建设。优化师资力量配备，新招聘教师1200人。研究实施基础教育综合改革政策，大力发展民办教育，抓好校长职级制改革试点。深化职业教育创新试验改革，力争职教公共实训中心一期建成使用。强化校车、校园、餐桌安全监管，确保师生安全。

健康养老提升工程。深化医药卫生体制改革，巩固完善基本药物制度，全面实施县级公立医院综合改革，推进基本公共卫生计生服务均等化。完成市人民医院整体规划和新门诊楼主体工程，开工建设生态科技城中心医院。实施县域医疗服务能力提升工程，加强重点学科建设，提高乡镇卫生院医疗服务水平。大力发展中医药事业，鼓励社会资本办医，形成多元化办医格局。大力发展养老服务产业，编制养老事业和产业发展规划，筹备建设市级颐养中心，新建城市社区日间照料中心57个、农村幸福院32个，新建、改造养老床位9000张，争取实现城市社区全覆盖、农村社区半覆盖。鼓励社会力量兴办养老服务机构，规划建设太阳庄园银龄公馆，满足多样化养老服务需求。

社会保障提升工程。启动全民参保登记计划，扩大社保覆盖面，基本实现全民养老和全民医保。巩固城乡居民基本养老、医疗保险制度整合成果，提高医保补贴标准和报销比例，完善居民大病保险政策。推进机关事业单位养老保险、工资制度改革，实施县以下机关公务员职务与职级并行、职级与待遇挂钩制度。健全“8+1”阳光救助体系，加快市残疾人康复中心和县级社会福利中心建设。着力提高弱势群体保障标准，城市低保标准提高到每人每月380至400元，农村低保标准提高到每人每年不低于2600元；农村五保集中、分散供养标准分别提高到每人每年4300元、3300元。加强农村精准扶贫开发，完成8万贫困人口扶贫任务。开工建设保障性住房16380套，基本建成8000套。

文化惠民提升工程。围绕打造区域文化高地，深入挖掘“德文化”、董子文化资源，大力弘扬运河文化、黄河文化、大禹文化，提升文化软实力。加强城乡四级公共文化设施建设，完善政府购买公共文化服务长效机制。实施好农村电影放映、农家书屋、实体书店等文化惠民项目，全年送戏下乡300场。开展“乡村记忆”工程，抓好文化遗产保护，加快推进运河主城区段保护开发规划修编和实施，支持黄河故道古桑树群申报世界重要农业文化遗产。大力发展文化产业，支持重点文化企业做大做强，建好文化园区、示范基地等平台。加快文化博览园等重大文化旅游创意项目招引和建设，开展等级景区和乡村旅游示范点创建工作，推动文化旅游融合发展。组织开展全民健身活动，办好全市第五届运动会。稳定适度低生育水平，促进人口均衡发展。高度重视双拥共建、国防动员、人民防空和民兵预备役工作，支持人武系统规范化建设。依法保障妇女、儿童、青少年、老年人、残疾人权益，认真做好民族宗教、外事侨务、对台事务、档案、史志等工作。

社会治理提升工程。加快市信访接待中心和信访信息化建设，推进领导干部公开接访，健全完善群众诉求表达、利益协调、权益保障和重大事项维稳风险评估机制。深入推进平安德州建设，抓好“大数据警务云”试点，加快建设智能交通安全系统。完善立体化治安防控体系，严厉打击各类违法犯罪活动，重点抓好小案侦防等涉及群众切身利益的问题。建立社会组织监督执法体系，加强社会组织培育管理。构建“网格化”、“实名制”的安全生产责任体系，强化隐患排查整治，防止重特大事故发生。加强食品药品监管，完善农产品追溯体系，重点治理“餐桌污染”，确保群众舌尖上的安全。

(六)突出改革创新，全面提升法治政府建设水平。深入贯彻依法治市基本方略，制定法治政府建设实施规划，加快政府职能转变，创新行政管理方式，确保各项工作在法治轨道上有序有效运行。

完善制度，推进依法行政。坚持讲法治、讲程序，注重用法治思维、手段和方式调节经济运行、管理社会事务。严格执行重大决策程序规定，完善政府法律顾问制度，健全重大决策终身责任追究制度及责任倒查机制。深化行政执法体制改革，推进食品药品、工商、质监、农业、环保等领域综合执法，探索在县级设立统一的综合执法机构。健全执法行为法制监督体制机制，积极推动省行政执法监督试点工作 礼拜五秘书网。认真执行市人大及其常委会的决议、决定，坚持重大事项向人大报告、向政协通报制度，主动接受人大法律监督和政协民主监督。健全人大、政协建议提案办理机制，主动服务人大和政协组织的视察、检查及调研活动，充分发挥代表、委员对政府工作的督导促进作用。多渠道听取各民主党派、工商联、无党派人士和各人民团体的意见建议。深化政务信息公开，加大电子政务建设力度，方便群众了解政府决策、监督政府工作。

简政放权，优化营商环境。严格落实行政审批事项目录清单、行政权力清单、政府责任清单，探索制定市场准入负面清单，编制权力运行流程图。继续压减行政审批事项，大幅削减隐性附着在审批中的前置条件和服务事项。全面实施流程再造，减少环节，压缩时限，降低收费。强化中介机构规范管理，实现与挂靠行政事业单位脱钩，加大政府向社会购买服务力度。加快事业单位分类改革，基本完成经营类事业单位转企改制任务。提升政务服务中心功能，深化“两集中、两到位”，实行“一窗受理、分散审核、同步审批、限时办结”模式。探索建立区域性技术评价机制，试点功能园区建设项目、成方连片出让土地区域化评估评审，促进投资创业便利化。

转变作风，倡导为民务实。持之以恒地贯彻中央八项规定精神，抓好群众路线教育实践活动整改落实，健全反对“四风”长效机制。充分发挥“12345”市民热线综合平台功能，提高问题解决率和群众满意率。广泛开展社情民意调查，增加老百姓的话语权和评判权。围绕重大发展问题，加强调查研究，搞好战略谋划，突出改革创新和重点项目引领，高起点、高标准编制“十三五”规划。健全目标责任清晰、监督考核有力的工作执行体系，实施重大事项、重点项目清单式管理，强化过程控制、社会监督，定期向全社会亮进度、晒问题，确保“实”字当头、“干”字为先。

敬畏法纪，恪守廉洁从政。严格落实党风廉政建设主体责任，认真履行“一岗双责”，严肃查处各类违纪违法案件，不断完善惩治和预防腐败体系。健全行政权力监督制约机制，突出国有资产、政府采购、公共资源、公共工程等重点领域，整合公共资源交易服务职能，强化内部流程控制，加强行政监察、审计监督，做到行政权力运行到哪里，监督就延伸到哪里。深入推进财税体制改革，加快构建全面规范、公开透明的预算制度，严格预算硬约束，加强预算绩效管理，市县两级政府及所有使用财政资金的部门均公开预决算。按照严控增量、消化存量的原则，规范政府债务管理，切实防范化解财政金融风险。大力倡导勤俭节约，坚决反对奢侈浪费，全面落实公务接待、办公用房、公共机构节能等制度，完成公务用车制度改革，确保“三公”经费只减不增，以为民务实清廉的优良作风取信于民、保障发展、维护稳定。

各位代表!改革发展任务艰巨，民生福祉责任重大。让我们在中共德州市委的正确领导下，紧紧依靠全市人民，勇于担当，务实苦干，为推进科学跨越发展、建设幸福德州做出新的更大贡献!
